# Elaboration and Characterization of Active Films Containing Iron–Montmorillonite Nanocomposites for O_2_ Scavenging

**DOI:** 10.3390/nano9091193

**Published:** 2019-08-23

**Authors:** Erland-Modeste Kombaya-Touckia-Linin, Sébastien Gaucel, Moulay T. Sougrati, Lorenzo Stievano, Nathalie Gontard, Valérie Guillard

**Affiliations:** 1 UMR, Ingénierie des Agropolymères et Technologies Emergentes, INRA, Univ. Montpellier, Montpellier SupAgro, CIRAD, 34060 Montpellier, France; 2 Institut Charles Gerhardt Montpellier, Univ. Montpellier, CNRS, 34090 Montpellier, France

**Keywords:** iron nanoparticles, iron boride, montmorillonite, nanocomposites, oxygen adsorption kinetics, modeling, ^57^Fe Mössbauer spectroscopy

## Abstract

Iron particles of sizes between 6 and 20 nm forming aggregates of 57 ± 17 nm were synthesized by chemical reduction of iron precursors on the surface of montmorillonite (MMT). This active MMT-Fe powder was then uniformly distributed in a linear low-density polyethylene (LLDPE) matrix by extrusion at atmospheric conditions, as confirmed by wide-angle X-ray scattering (WAXS), which also detected a partial exfoliation of the nanoclays. Thermogravimetric analysis (TGA) did not detect any significant modification of the degradation temperature between nanocomposites and active nanocomposites. ^57^Fe Mössbauer spectroscopy evidenced the formation of a majority of iron boride in MMT-Fe as well as in the active film containing it. The LLDPE.Fu15.MMT-Fe3.75 and LLDPE.Fu15.MMT-Fe6.25 films had oxygen-scavenging capacities of 0.031 ± 0.002 and 0.055 ± 0.009 g(O_2_)/g(Fe), respectively, while the neat powder had an adsorption capacity of 0.122 g(O_2_)/g(Fe). This result confirms that the fresh film samples were partially oxidized shortly after thermomechanical processing (60% of oxidized species according to Mössbauer spectroscopy). No significant difference in oxygen permeability was observed when MMT-Fe was added. This was related to the relatively small film surface used for measuring the permeability. The reaction–diffusion model proposed here was able to reproduce the observed data of O_2_ adsorption in an active nanocomposite, which validated the O_2_ adsorption model previously developed for dried MMT-Fe powder.

## 1. Introduction

Much attention is currently being paid to the development of O_2_ barrier monolayer films that are as performing as the multilayer complex materials currently used for commercial applications and can still maintain the end of life characteristics of a monolayer pure matrix (recyclability, biodegradability, etc.). Indeed, multilayer packaging materials that nowadays offer the required protection against O_2_ and oxidation are difficult to recycle due to their complex formulations and thus have very high environmental impact [1,2,3]. Moreover, they are expensive and represent an economic burden for the food industry.

To obtain monolayer films with good O_2_ barrier properties, one strategy is to develop materials based on nanocomposites such as, for instance, clay–polymer nanocomposites. Thanks to the dispersion of nanoscale clays in the bulk of the polymer, a significant decrease of O_2_ permeability can be achieved compared with that of a pure polymer matrix [4,5,6]. This is observed when a highly exfoliated structure is obtained, that is, when the polymer penetrates into the layered structure of the clay platelets, taking them apart and leading to individual clay foils randomly dispersed in the polymer. The observed improvement in barrier properties can be ascribed to the extension of the length and tortuosity of the diffusing gas flow paths, which leads to a reduction of the diffusivity of the gas through the polymer nanocomposite [6,7,8]. In practice, however, highly exfoliated structures are difficult to achieve, and the expected decrease in O_2_ permeability is not always as important as expected, as recently explained by Wolf et al. [6].

To improve the O_2_ barrier performance of monolayer films, another strategy relies on the introduction of active agents reacting with the O_2_ that permeates from the surroundings [7,8,9]. The most widely used O_2_ scavengers are based on powders containing polyamide, sulfite, and iron species. Among iron-based systems, zerovalent iron nanoparticles (ZVINPs) have recently gained attention for their application in active packaging. ZVINPs are used for their very high surface reactivity in environmental applications (remediation of inorganic contaminants in water and soil, e.g., U, Cd, Zn, NO_3_^−^, etc.) [10,11,12,13] and medical applications (detoxification of biological fluids, tissue healing, etc.) [14]. Their use in packaging applications to produce materials with active oxygen-scavenging properties is much more recent and still less studied. A previous work from Kombaya-Touckia-Linin et al. (2019) [15] recently demonstrated the feasibility of synthesizing via a liquid route low-size spherical ZVINPs of about 50 nm in diameter directly on a nanoclay support. These authors provided a thorough characterization of their iron-based nanoscale oxygen scavengers, including full characterization of the O_2_ adsorption kinetics. The as-synthetized active powder was found to have an adsorption capacity of 122 mg of O_2_ per gram of absorber (91.7 mL of O_2_ per gram of iron). The main advantage of using ZVINPs synthetized on nanoclay supports is that they can be easily included in the bulk polymer using classical thermomechanical processes [15,16]. In such composites, both a passive barrier (provided by the tortuosity induced by nanoclays) and an active effect (due to O_2_-scavenging compounds) are expected with enhancement of the global performance of the material. Moreover, ZVINPs in such composites are entrapped in the bulk polymer and thus do not have a potential mobility to migrate into food preventing human exposure when used as food contact materials. It is generally assumed that consumers will not be exposed to nanoparticles from food contact polymers as long as their size exceeds 4 nm in at least one of their dimensions and the contact surface is not altered by mechanical surface stress during application [17].

Only three articles could be found in the scientific literature dealing with active nanocomposites with ZVINPs as active oxygen scavengers: that of Khalaj et al. [18], who prepared nanocomposite polypropylene films containing ZVINPs anchored on montmorillonite (MMT) via a thermomechanical process; the work of Foltynowicz et al. [7], who synthesized ZVINPs and dispersed them in a silicon matrix; and the paper of Busolo and Lagaron [9], who evaluated the oxygen-scavenging potential of a synthetic ZVINPs-containing kaolinite for food packaging plastics. In all these studies, ZVINPs are able to intercept and trap oxygen with adsorption capacities varying from 2.3 to 4.3 mL O_2_ per gram of active film composite. However, in all these previous studies, the lack of full structural characterization of these ZVINPs systems did not permit the authors to carry out deep, mechanistic analyses of the adsorption of O_2_ and to provide a precise relationship between iron speciation and the observed adsorption properties.

The current work had the original ambition of describing, from a mechanistic point of view, the phenomenon of O_2_ diffusion–reaction within an active film containing nanoscale iron-based oxygen scavengers. This goal was sought via the production of active nanocomposites containing well-defined and characterized ZVINPs supported on nanosized MMT-Fe according to the MMT-Fe synthesis previously set up by Kombaya-Touckia-Linin et al. (2019) [15]. These nanoparticles were dispersed in a linear low-density polyethylene (LLDPE) matrix via an extrusion process. LLDPE was chosen as a model polymer since it is widely used in the packaging industry and its thermal, mechanical, and barrier properties are well known. Its processing with nanoclays using classical thermomechanical processes is well characterized too, and the exfoliation of MMT is well mastered, especially when polyethylene-grafted maleic anhydride (PE-g-MA) is added to improve the compatibility between the clay and polymer matrix [19,20]. The synthesized composites were characterized by transmission electron microscopy (TEM), wide-angle X-ray scattering (WAXS), differential scanning calorimetry (DSC), thermogravimetric analysis (TGA), and ^57^Fe Mössbauer spectroscopy. The oxygen adsorption rate and maximal adsorption capacity were experimentally measured and correlated with the iron speciation in the film and its evolution with time. The oxygen adsorption kinetic was successfully modeled using a two-species kinetic reaction. Finally, the O_2_ permeability of the final materials was also evaluated.

## 2. Materials and Methods

### 2.1. Materials

LLDPE (LL 1002YB melt flow index 2.0 g.10 min^−1^, density 0.918 g·cm^−3^) was supplied by Exxon Mobil Chemicals (Germany). MMT naturally modified by alkyl ammonium (Cloisite 20A: CEC of 95 meq·100 g^−1^ clay mineral and a basal distance value d_001_ = 2.65 nm) was obtained from Southern Clay Products Inc.

LDPE-g-MA (Fusabond E226: maleic-anhydride-modified polyethylene with a melt flow index of 1.75 g·10 min^−1^ and density of 0.93 g·cm^−3^), used to enhance the compatibility between the nanoclay and polymer matrix, was kindly provided by Dupont™ (Germany).

High-purity reagents, including ethanol (99%), NaOH (32% aqueous solution), FeCl_3_·6H_2_O (98%), and sodium borohydride (NaBH_4_), were purchased from Sigma-Aldrich (Germany) and used as received. Distilled and demineralized water was used throughout these experiments.

#### 2.1.1. Synthesis of MMT-Fe

Montmorillonite-supported zerovalent iron particles (MMT-Fe) were obtained through the liquid route by the reduction of ferric iron with sodium borohydride according to a previous work [15]. Briefly, for the preparation of each batch, 10.1 g of FeCl_3_·6H_2_O was dissolved in 500 mL of a solution containing 1:4 v/v of deionized water and absolute ethanol. MMT (10.5 g) was added to the FeCl_3_ solution and the suspension was stirred for 40 h at room temperature.

The iron-reducing solution (7.5 g of NaBH_4_ in 500 mL of a basic aqueous solution made of 475 mL of deionized water and 25 mL of 32% NaOH) was stirred for 16 h. Use of a basic solution to dissociate sodium borohydride prevents the production of hydrogen [21]. The FeCl_3_-MMT suspension and the NaBH_4_ solution were both degassed in flowing argon for 2 h to remove the dissolved oxygen.

Dropwise, 250 mL of the NaBH_4_ solution were added to the FeCl_3_-MMT suspension under vigorous stirring (400 rpm). The molar ratio of borohydride to Fe was 4:1. Borohydride is expected to reduce Fe(III) to Fe(0) according to Equation (1):

(1) $$\mathrm{Fe}{\left(H_{2}O\right)}_{6}^{3+}+3{\mathrm{BH}}_{4}^{-}+3H_{2}O\to {\mathrm{Fe}}^{0}+3B{\left(\mathrm{OH}\right)}_{3}+10.5H_{2}.$$

After the addition of NaBH_4_, the solution turned black, indicating the reduction of ferric iron. Synthesis was done at room temperature. A continuous argon flow was used in the reactor to remove the oxygen. A constant stirring speed was maintained throughout the synthesis.

The solid recovered by filtration was washed three times in absolute ethanol (99%), dried in flowing argon for 24 h at room temperature, and then stored in anoxic conditions before use for a maximum of one month before disposal. The nanostructured MMT-Fe final product was used for active nanocomposite design.

#### 2.1.2. Processing of MMT-Fe/LLDPE Films

The active polyolefin composites were prepared by mixing a masterbatch of LLDPE containing either 9 or 15 wt % of LLDPE-g-MA with 3.75 or 6.25 wt % of MMT-Fe, respectively, using a corotating twin-screw extruder (Thermo Scientific™ EuroLab 16, Germany) in atmospheric conditions with a L/D ratio of 40 and a screw diameter of 16 mm at a screw speed of 200 rpm and feed rate of 1.0 kg·h^−1^. The temperature profile of the extruder from the feeder to the die increased regularly from 160 to 180 °C, as described in detail elsewhere [19,20]. The maximum temperature was set at 180 °C to prevent decomposition of the modified MMT. Control samples containing pure LLDPE as well as samples containing iron-free MMT were also prepared in the same conditions. No specific precautions were taken to protect the active material in order to mimic the processing conditions of the industrial operation environment. The extruder was filled regularly and continuously with a very small quantity of active components to avoid its overexposure to atmospheric O_2_ in the feeder (<1 min in the presence of atmospheric O_2_). Uniform thin films were produced using a flat die at the end of the extruder, which was followed by calendaring. The as-produced active films took less than 10 min to be cold, cut, and stored in anoxic conditions. Composition and labeling of the prepared active films are summarized in Table 1.

### 2.2. Methods

#### 2.2.1. Structural and Morphological Analysis

For TEM analyses, thin sections (100 nm) of the prepared films obtained by ultramicrotomy under cryogenic conditions (−100 °C) were deposited on a holey carbon film supported on a 300-mesh copper TEM grid and analyzed using a Jeol 1200EX2 transmission electron microscope with an acceleration voltage of 100 kV.

WAXS experiments were performed at Laboratoire Charles Coulomb (Univ. Montpellier, France) using an in-house setup consisting of a high brightness and low power Cu Kα X-ray tube coupled with a spheric multilayer optic (GeniX^3D^ from Xenocs) delivering an ultralow divergent beam (0.5 mrad). Scatterless slits were used to produce a clean 0.8 mm diameter beam (35 Mphotons/s) at the sample stage. A transmission configuration was used and the scattered intensity was measured using a Schneider 2D image plate detector placed 0.2 m from the sample. The films were mounted on a custom-made sample holder having Kapton windows to protect the samples from ambient air.

All intensities were corrected by transmission and the empty holder contribution was subtracted. Interplanar distance (d) was calculated using Bragg’s equation: nλ=2dsinθ, where n is a positive integer, λ is the wavelength of the X-ray beam, and θ is the diffraction angle.

#### 2.2.2. Thermal Properties

TGA was performed using a TGA Q50 thermobalance (TA Instruments) with a heating ramp of 10 °C·min^−1^ from room temperature to 1000 °C under flowing air (50 mL·min^−1^). Approximately 10 mg of the sample was analyzed in an open sapphire crucible. Thermal degradation was characterized by two temperatures: $T_{\mathrm{onset}}$ (beginning of the degradation process) and $T_{\mathrm{offset}}$ (end of the degradation process).

DSC measurements were carried out using a thermomodulated calorimeter (Q200 modulated DSC, TA Instruments). The sample films (around 7–10 mg) were placed in an aluminum pan and heated from 30 to 200 °C at a heating rate of 10 °C·min^−1^ and held at this temperature for 5 min to erase the thermal history. The samples were then cooled to room temperature with a cooling rate of 10 °C·min^−1^. Crystallization and melting thermograms of the samples were obtained. From the DSC thermograms, the melting temperature ($T_{m}$) and heat of fusion ($\Delta H_{m}$) were obtained. The degree of crystallinity $x_{c}$ was then calculated as follows:

(2) $$x_{c}=\frac{\Delta H_{m}+\Delta H_{c}}{\Delta H_{m}^{0}}\;100$$

where $\Delta H_{m}$ and $\Delta H_{c}$ are the enthalpies of fusion and crystallization, respectively, in J·g^−1^, and $\Delta H_{m}^{0}$ is the enthalpy of fusion of fully crystalline polyethylene (293 J·g^−1^) [22].

#### 2.2.3. O_2_ and Vapor Barrier Properties

Oxygen permeability was determined according to the standard procedure DIN 53380-5 using an oxygen permeation cell based on an optical luminescence quenching method (OTR-Pst6, PresSens-GmbH) as described elsewhere [23,24]. Oxygen permeability, $P_{O_{2}}$, in mol·m^−1^·s^−1^·Pa^−1^, was calculated using the following equation:

(3) $$P_{O_{2}}=\frac{\dot{P}\;l}{A\;P_{\mathrm{atm}}}$$

where $\dot{P}$ (mol·s^−1^) is the slope of the increase of the oxygen partial pressure in the upper test chamber, l (m) is the average thickness of the film, A (m^2^) is the film area, and P_atm_ (Pa) is the standard atmospheric pressure of oxygen. The measurements were replicated four times. The thickness of the films was determined using a precision micrometer (Mitutoyo).

Water vapor permeability (WVP) (mol·m^−1^·s^−1^·Pa^−1^) measurements were performed by a gravimetric method [25]. Film discs were hermetically sealed (with Teflon seals) in a glass permeation cell containing distilled water and placed at 20 °C inside a desiccator with silica gel. The weight evolution was measured every 24 h for 7 days and plotted against time. The water vapor that transferred through the films was calculated by linear regression:

(4) $$\mathrm{WVP}=\frac{w\;L}{A\;\Delta_{p}}$$

where w (mol·s^−1^) is the slope of the weight loss versus time, L (m) is the film thickness at equilibrium measured at the end of the experiment, A (m^2^) is the area of exposed film, and $\Delta_{p}$ the differential vapor pressure of water at 20°C (2337 Pa).

#### 2.2.4. Oxygen-Scavenging Capacity

The oxygen-scavenging capacity of the nanocomposite films was obtained by measuring the time–O_2_ depletion curve in the headspace of a tightly closed reactor (at least five replications). To do that, 25–30 g of the sample was placed in the reactor, into which 10 mL of pure water was also poured at the bottom to achieve a saturated relative humidity (RH). The reactor was then closed and placed at 20 ± 1 °C in a temperature-controlled oven.

The O_2_ partial pressure in the reactor was monitored using a noninvasive optical oxygen sensor (Presens Precision Sensing, Germany) connected to an oxygen measuring device. The limit of detection was 0.03% and the accuracy of 0.4% O_2_ at 20.9% of O_2_. O_2_ partial pressure was recorded until scavenger saturation, which was achieved when no further O_2_ consumption was detected (constant O_2_ partial pressure in headspace). The mass of absorbed O_2_ (in grams per gram of iron) was calculated from the O_2_–headspace depletion curve using the perfect gas law. The procedure is well described in detail in the previous work of Kombaya-Touckia-Linin et al. [15].

#### 2.2.5. Iron Content by Inductively Coupled Plasma Optical Emission Spectrometry (ICP-OES)

The elemental iron content in MMT-Fe was obtained by ICP-OES (Agilent Technologies) on mineralized solutions of the samples previously obtained by fluoro-nitro perchloric etching according to standard procedures NF X31−147 and ISO 14869−1 [26,27].

#### 2.2.6. Mössbauer Spectroscopy

Transmission ^57^Fe Mössbauer spectra were collected with a ^57^Co:Rh source at room temperature. The samples were cooled at 5 K in a helium-flow cryostat (SHI-850 Series, Janis). The velocity driver was operated with a triangular velocity waveform. The velocity scale was calibrated at room temperature with the magnetically split sextet of a high-purity α-Fe foil. The spectra were fitted by least-squares methods to appropriate combinations of Lorentzian profiles representing the quadrupole doublets, magnetic sextets, or octets using the program PC-Mos II [28]. In this way, spectral parameters such as the hyperfine magnetic field (H) quadrupole splitting (Δ), the isomer shift (δ), and the line width at half-maximum (Γ) of the different spectral components were determined for the whole series of spectra. Isomer shifts are given relative to α-Fe at room temperature. To quantify the amount of iron within the films, 16 layers of film samples were mounted together with an iron reference of known iron content on the cryostat.

### 2.3. Modeling of Oxygen Adsorption in the Active Nanocomposite

#### 2.3.1. Modeling of O_2_ Adsorption for Iron Nanoparticles

An iron-based O_2_ scavenger was considered to be composed of iron nanoparticles supported on MMT clay nanoplatelets. The adsorption properties of this scavenger were characterized and formalized using a predictive model, as in the previous work of Kombaya-Touckia-Linin et al. [15]. The oxidation of both ${\mathrm{Fe}}^{0}$ and ${\mathrm{Fe}(\mathrm{OH})}_{2}$ species were simultaneously considered, each one following an independent second-order kinetic law. The oxidation model is summarized in Equation (5):

(5) $$\begin{array}{l} \frac{{\mathrm{dC}}_{O_{2}}}{\mathrm{dt}}{=-k}_{1}\;n_{1}\;C_{O_{2}}n_{\mathrm{Fe}}-k_{2}\;n_{2}\;C_{O_{2}}C_{{\mathrm{Fe}(\mathrm{OH})}_{2}}\\ \frac{{\mathrm{dC}}_{\mathrm{Fe}}}{\mathrm{dt}}=-k_{1}C_{O_{2}}C_{\mathrm{Fe}}\\ \frac{{\mathrm{dC}}_{{\mathrm{Fe}(\mathrm{OH})}_{2}}}{\mathrm{dt}}{=-k}_{2}C_{O_{2}}C_{{\mathrm{Fe}(\mathrm{OH})}_{2}} \end{array}$$

where $C_{O_{2}}$, $C_{\mathrm{Fe}}$, and $C_{{\mathrm{Fe}(\mathrm{OH})}_{2}}$ are the concentrations (mol·m^−3^) of $O_{2}$, Fe, and ${\mathrm{Fe}(\mathrm{OH})}_{2}$; $n_{1}$ and $n_{2}$ are the apparent stoichiometric coefficients for oxidation of Fe and ${\mathrm{Fe}(\mathrm{OH})}_{2}$ by O_2_; and $k_{1}$ and $k_{2}$ are the kinetic coefficients (m^3^·s^−1^·mol^−1^) for oxidation of Fe and ${\mathrm{Fe}(\mathrm{OH})}_{2}$, respectively.

#### 2.3.2. Modeling of O_2_ Adsorption for an Active Nanocomposite

The phenomena occurring at the nanocomposite level can be described by a reaction–diffusion system. Fick’s law of diffusion was applied to describe the diffusion of O_2_. The reaction part was modeled according to Equation (5).

MMT nanoplatelets and iron nanoparticles were assumed to be immobile in the polymer matrix, and the nanocomposite was considered as a homogenous material, that is, with a single apparent O_2_ diffusivity $D_{O_{2}}$(m^2^·s^−1^) and the iron species uniformly distributed in the material. Finally, the mathematical model for a plane film geometry reduced to the one-dimensional reaction–diffusion system is given in Equation (6), for x∈]−L/2, L/2[, where L is the thickness of the film:

(6) $$\begin{array}{l} \frac{\partial C_{O_{2}}\left(t,x\right)}{\partial t}=D_{O_{2}}\;\frac{\partial^{2}C_{O_{2}}\left(t,x\right)}{\partial x^{2}}-k_{1}n_{1}C_{O_{2}}\left(t,x\right)C_{\mathrm{Fe}}\left(t,x\right)-k_{2}n_{2}C_{O_{2}}\left(t,x\right)C_{\mathrm{Fe}{\left(\mathrm{OH}\right)}_{2}}\left(t,x\right)\\ \frac{\partial C_{\mathrm{Fe}}\left(t,x\right)}{\partial t}=-k_{1}C_{O_{2}}\left(t,x\right)C_{\mathrm{Fe}}\left(t,x\right)\\ \frac{\partial C_{\mathrm{Fe}{\left(\mathrm{OH}\right)}_{2}}\left(t,x\right)}{\partial t}=-k_{2}C_{O_{2}}\left(t,x\right)C_{\mathrm{Fe}{\left(\mathrm{OH}\right)}_{2}}\left(t,x\right) \end{array}$$

with the initial (Equation (7)) and boundary (Equation (8)) conditions given hereafter.

Initial condition: at initial time $t_{0}$, uniform concentrations $C_{\mathrm{Fe}}\;\left(t_{0}\right)$, and $C_{{\mathrm{Fe}(\mathrm{OH})}_{2}}\left(t_{0}\right)$ were considered and computed according to Equation (7):

(7) $$\begin{array}{c} C_{\mathrm{Fe}}\;\left(t_{0}\right)=\frac{\rho_{f}\;x_{F_{e}}^{f}\;x_{F_{e}}}{M_{F_{e}}}\\ C_{{\mathrm{Fe}(\mathrm{OH})}_{2}}\left(t_{0}\right)=\frac{\rho_{f}\;x_{F_{e}}^{f}\;x_{{\mathrm{Fe}(\mathrm{OH})}_{2}}}{M_{F_{e}}} \end{array}$$

where $x_{F_{e}}^{f}$(kilograms of iron per kilogram of sample) is the mass fraction of iron in the sample, $x_{F_{e}}$ and $x_{{\mathrm{Fe}(\mathrm{OH})}_{2}}$(kilograms per kilogram of total iron) are the mass fraction of zerovalent and divalent iron, respectively, $M_{F_{e}}$(kg·mol^−1^) is the molar mass of iron, and $\rho_{f}$ (kg·m^−3^) is the apparent density of the nanocomposite. A null oxygen concentration, $C_{O_{2}}\left(t_{0}\right)=0$, was assumed.

Boundary condition: the external conditions at the upper and lower surfaces of the active nanocomposite film surrounded by headspace were equal. For instance, the flow that went in the film at the upper surfaces $\Gamma_{L/2}$ is given by

(8) $$\varphi_{L/2}=k\;A\;\left(C_{0_{2},\;\mathrm{HS}}-C_{0_{2},\;\mathrm{HS},L/2}\right)$$

where k (m·s^−1^) is the external mass transfer coefficient; A (m^2^) is the surface area of the headspace–film interface, which was assumed to be identical for the upper and lower surfaces; and $C_{0_{2},\;\mathrm{HS}}$ (mol·m^−3^) and $C_{0_{2},\;\mathrm{HS},i}$ (mol·m^−3^) are, respectively, the O_2_ concentration in the headspace and at the vicinity of the composite surface. Converting concentration into partial pressure thanks to the ideal gas law and Henry’s law (more details in Appendix A) finally leads to the two following boundary conditions:

(9) $$\begin{array}{l} D_{O_{2}}\;\frac{\partial C_{O_{2}}\left(t,x\right)}{\partial x}=\frac{\varphi_{\frac{L}{2}}}{A}=\frac{k}{\mathrm{RT}}\;\left(p_{0_{2},\;\mathrm{HS}}-\frac{C_{O_{2}}\left(t,x\right)}{k_{H}}\right)\;\mathrm{at}\;x=\frac{L}{2}\;\mathrm{and}\;\forall t\geq 0\\ D_{O_{2}}\;\frac{\partial C_{O_{2}}\left(t,x\right)}{\partial x}=\frac{\varphi_{-\frac{L}{2}}}{A}=\frac{k}{\mathrm{RT}}\;\left(p_{0_{2},\;\mathrm{HS}}-\frac{C_{O_{2}}\left(t,x\right)}{k_{H}}\right)\;\mathrm{at}\;x=-\frac{L}{2}\;\mathrm{and}\;\forall t\geq 0. \end{array}$$

The external mass transfer coefficient k can be expressed through the dimensionless Biot number Bi, as presented in Equation (10):

(10) $$k=\frac{2\;\mathrm{Bi}\;D_{O_{2}}}{L}.$$

Mass balance of O_2_ in the headspace: the composite film was assumed to be inside an airtight container, so that the headspace volume $V_{\mathrm{HS}}$ (m^3^) was constant and the gas flow through the container was negligible. Consequently, the variation of the headspace O_2_ partial pressure could be computed by integrating the flows $\varphi_{L/2}$ and $\varphi_{-L/2}$ on the film surface area and again using the ideal gas law, which leads to Equation (11):

(11) $$\frac{\partial p_{0_{2},\;\mathrm{HS}}}{\partial t}=\frac{\mathrm{RT}}{V_{\mathrm{HS}}}\left(\iint_{\Gamma_{L/2}}\varphi_{L/2}\mathrm{dS}+\iint_{\Gamma_{-L/2}}\varphi_{-L/2}\mathrm{dS}\right).$$

As the flows are homogeneous on the surface, Equation (11) is rewritten as

(12) $$\frac{\partial p_{0_{2},\;\mathrm{HS}}}{\partial t}=k\frac{A}{V_{\mathrm{HS}}}\left(2\;p_{0_{2},\;\mathrm{HS}}-\frac{C_{O_{2}}\left(t,x=L/2\right)}{k_{H}}-\frac{C_{O_{2}}\left(t,x=L/2\right)}{k_{H}}\right).$$

#### 2.3.3. Numerical Simulations

Numerical simulations were performed with a high Biot number (Bi = 10^4^), characteristic of this diffusion-limited case. The partial differential equation system (Equations (2), (3), (8), and (11)) was transformed into an ordinary differential equation (ODE) system by spatial discretization with a second-order central difference method and a mesh of 100 nodes. The resulting ODE system was numerically solved using MATLAB (MathWorks).

### 2.4. Statistics

Statistical tests were performed using R software for statistical computing (R, 2014). One-way ANOVA tests were performed to verify if the variances of the datasets were statistically different or not. Comparisons between the compositions were performed by pairwise comparisons using Tukey’s test. Different letters were used to denote significant differences between datasets (level of significance α = 0.05, unless stated).

## 3. Results

### 3.1. Structural and Morphological Characterization of the MMT-Fe and MMT-Fe/LLDPE Films

The as-synthesized MMT-Fe powder contained about 0.22 ± 0.01 g of Fe per gram of powder, as determined by ICP-OES. This powder was embedded, after a short storage period in anoxic conditions (15 days max.), in a matrix of LLDPE containing PE-g-MA (15 wt %) as an additive in quantities previously tested and validated [19,20,29].

**Figure 1:**
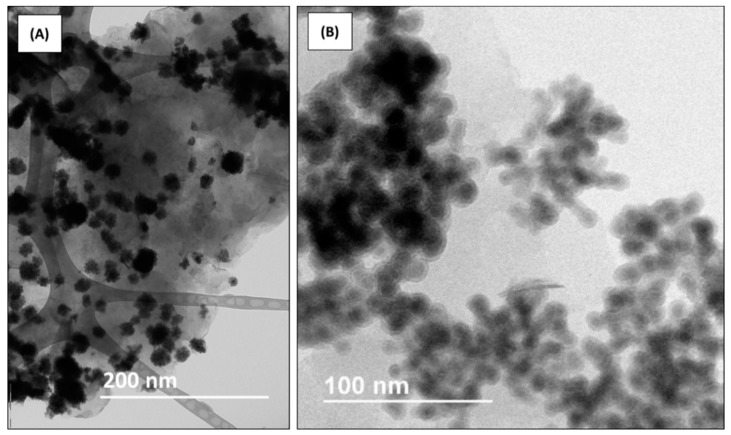
Transmission electron microscopy (TEM) images of MMT-Fe at different magnifications, showing the dispersion of the aggregates (**A**) and their microstructure (**B**).

TEM images of the MMT-Fe particles are shown in Figure 1. Particles of sizes between 6 and 20 nm forming aggregates of 57 ± 17 nm [15] were observed on the surface of montmorillonite.

**Figure 2:**
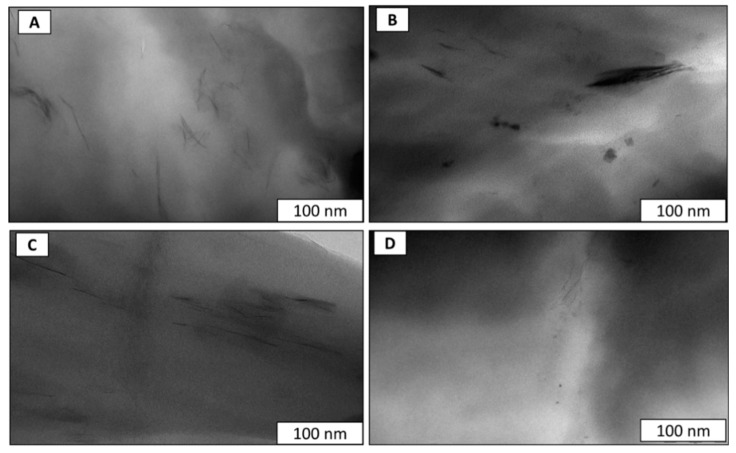
TEM micrographs of (**A**) LLDPE.Fu15.MMT5 film, (**B**) LLDPE.Fu15.MMT-Fe6.25 film, (**C**) LLDPE.Fu15.MMT3 film, and (**D**) LLDPE.Fu15.MMT-Fe3.75 film.

TEM analyses were also performed to evaluate the dispersion of MMT and MMT-Fe in the LLDPE matrix. The micrographs shown in Figure 2 show a quite good dispersion of the MMT in all the composites, even though a few small agglomerates of clay platelets were observed in LLDPE/MMT and LLDPE/MMT-Fe. A preferred orientation of the nanoclay platelets was observed for all nanocomposites, with the exception of the nanocomposite with high MMT loading. This effect may have been related to the shear forces induced during extrusion and calendaring, which aligned the nanoplatelets in the direction of the flow. The presence of iron nanoparticles was not clearly visible from TEM pictures except at a high MMT-Fe content, where some iron aggregates are visible in the TEM picture (Figure 2B).

**Figure 3:**
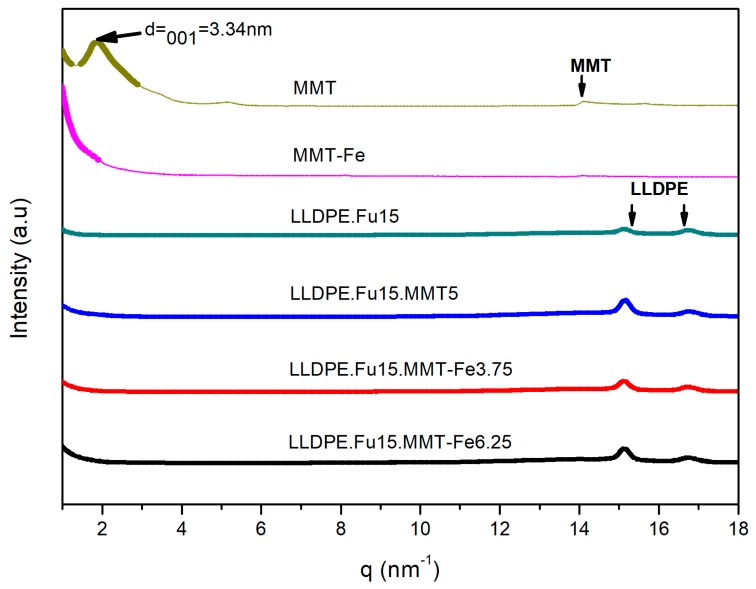
Wide-angle X-ray scattering (WAXS) patterns for nanoclay powder MMT, MMT-Fe, LLDPE, and LLDPE matrix compatibilized with PE-g-MA and active nanocomposite films (the curves are vertically offset).

The WAXS results, shown in Figure 3, confirmed that the nanoclays were partially exfoliated in the polymer independent of the formulation and of the presence of the active compounds. The WAXS pattern of MMT showed an intense diffraction peak, which corresponded to a spacing distance ($d_{001}$) equal to 3.34 nm [30]. This peak was no longer observed on patterns of MMT-Fe and the active nanocomposites, indicating a virtually complete delamination of the clay in these materials.

### 3.2. Effect of MMT-Fe Content on the Thermal Stability of LLDPE Nanocomposite Films

**Figure 4:**
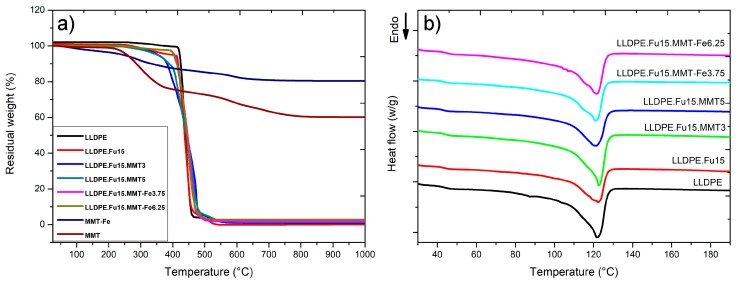
(**a**) Thermogravimetric (TG) curves in flowing air of LLDPE, MMT, MMT-Fe, and nanocomposites with different MMT and MMT-Fe contents. (**b**) Differential scanning calorimetry (DSC) thermograms of LLDPE and LLDPE nanocomposites.

The results of DSC and TGA, which were used to study the thermal stability of MMT-Fe and the active films, are shown in Figure 4. The corresponding data (heat of fusion, melting point, and crystallinity determined by DSC and temperature of degradation determined by TGA) are compiled in Table 2. For MMT and MMT-Fe, a first mass loss was observed around 84 °C, corresponding to the desorption of volatile species (ethanol), while a second mass loss between 200 and 500 °C, characteristic of Cloisite 20A, corresponded to the departure of organic substances (alkyl ammonium). No significant difference was observed by TGA between the different polymer nanocomposites: the presence of the PE-g-MA, MMT, and/or MMT-Fe had no significant influence on the thermal degradation of the polymer matrix, which decomposed at 450 °C in all of studied materials.

DSC was used to study the influence of MMT and MMT-Fe on melting temperature (Tm), specific melting enthalpy (ΔHm), and the degree of crystallinity of LLDPE (Table 2). No significant differences in the melting temperatures were observed for LLDPE, LLDPE.Fu15, and its active nanocomposite samples (LLDPE.Fu.MMT3, LLDPE.Fu15.MMT-Fe3.75, and LLDPE.Fu15.MMT-Fe6. 25). Similar results were obtained for the heat of fusion and crystallinity, except for LLDPE.Fu15.MMT5, which displayed significantly lower crystallinity. We can confirm from these results that the MMT and MMT-Fe powder did not act as nucleating agents for the LLDPE pure matrix.

### 3.3. Mössbauer Spectroscopy Study of the Active Nanocomposite

**Figure 5:**
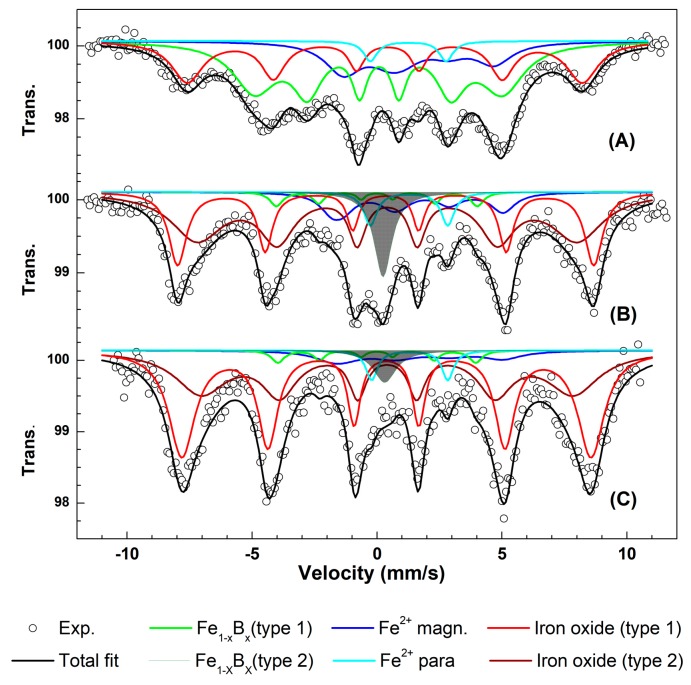
Examples of ^57^Fe Mössbauer spectra at 5 K for (**A**) MMT-Fe, (**B**) fresh LLDPE.Fu15%.MMT-Fe6.25%, and (**C**) LLDPE.Fu15%.MMT-Fe6.25% exposed to ambient air for four months.

The ^57^Fe Mössbauer spectra at 5 K of the MMT-Fe powder, the fresh film obtained directly after extrusion (LLDPE.Fu15.MMT-Fe6.25), and the same sample exposed to ambient air for four months are shown in Figure 5. The spectra were fitted to several spectral components as described in Kombaya-Touckia-Linin et al. [15]. The same analysis was performed on similar samples based on LLDPE.Fu15.MMT-Fe3.75.

In the spectrum of MMT-Fe, four different iron environments can be detected: magnetically ordered Fe^2+^ (blue octet), paramagnetic Fe^2+^ (cyan doublet), Fe^3+^ oxide (red sextet), and the blue sextet characterized by an isomer shift of 0.27 mm/s and a hyperfine field of 30.7 T indicating the presence of Fe_1−X_B_X_, as already observed previously [15,31]. These parameters, significantly different from those of α-Fe metal, confirm the formation of amorphous iron boride from the reduction of iron by the borohydride anion [32,33]. Fe_1−X_B_X_ was also detected on the fresh films LLDPE.Fu15.MMT-Fe6.25 and LLDPE.Fu15.MMT-Fe3.75. In the films, however, this sextet was no longer detected and was replaced by a large paramagnetic singlet, probably resulting from the film processing treatment, which could have induced structural disorder in Fe_1−x_B_x_. None of the observed Fe_1−x_B_x_ components were stable in air and were rapidly converted to iron oxides upon air exposure.

For the fresh LLDPE.Fu15.MMT-Fe6.25 film, the two dominant components were typical of Fe^3+^ in iron oxides or oxyhydroxides (Table 3), summing up to 75% of the total resonance area. This confirms that MMT-Fe was already partially oxidized during the film processing step. The presence of two large sextets indicated the presence of a distribution of hyperfine magnetic fields, typical of relatively ill-defined oxide or oxyhydroxide species. For LLDPE.Fu15.MMT-Fe6.25 exposed to air for four months, an increase of the oxide resonance area to 86% was observed, confirming the gradual oxidation of the sample with time.

### 3.4. Oxygen-Scavenging Capabilities for MMT-Fe and Active Nanocomposites

**Figure 6:**
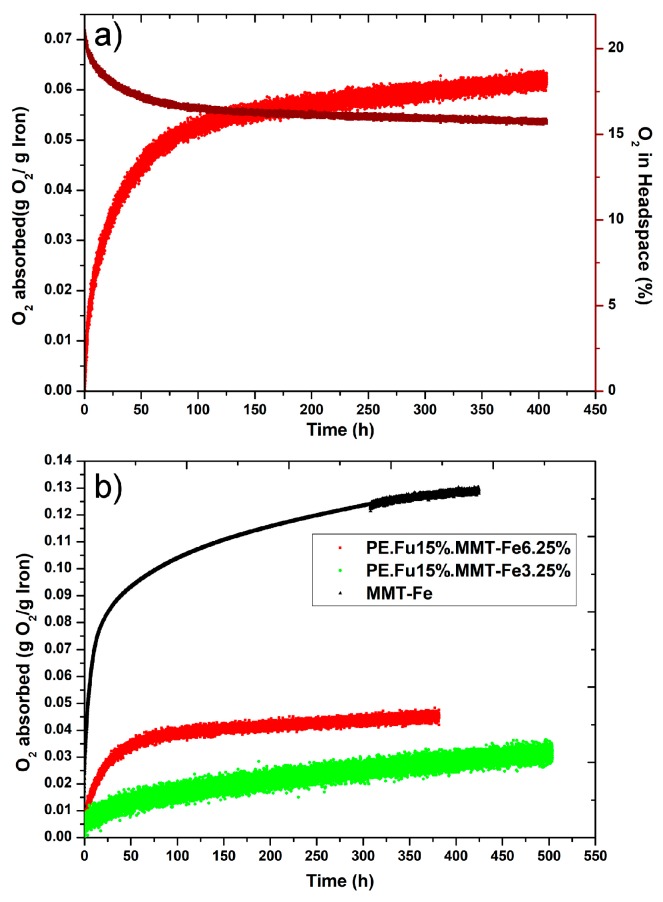
(**a**) Example of experimental O_2_–headspace depletion curves and corresponding O_2_ adsorption curves for LLDPE.Fu15.MMT-Fe6.25 and (**b**) comparison of oxygen adsorption of dried MMT-Fe powder, LLDPE.Fu15.MMT-Fe3.75, and LLDPE.Fu15.MMT-Fe6.25 films.

The oxygen adsorption capacity of the synthesized MMT-Fe powder and the films was measured in static conditions in a saturated water vapor atmosphere (100% relative humidity), as the oxidation of iron by oxygen requires the presence of water molecules (0.5 g of water·g^−1^ of iron) [34,35]. At least five replications for each sample were carried out. Figure 6a shows the headspace–O_2_ depletion curve and the derived O_2_ adsorption curve (calculated by using the perfect gas law approximation) for the active film LLDPE.Fu15.MMT-Fe6.25.

The adsorption capacity of the films (LLDPE.Fu15.MMT-Fe6.25 and LLDPE.Fu15.MMT-Fe3.75) was compared with that of MMT-Fe (Figure 6b). The film adsorption capacity remained low compared with that of the powder used alone. In fact, oxygen-scavenging capacities of 31 ± 2 and 55 ± 9 mg O_2_·g^−1^ iron were obtained for LLDPE.Fu15.MMT-Fe3.75 and LLDPE.Fu15.MMT-Fe6.25, respectively, while the adsorption capacity of MMT-Fe was on average 122 mg O_2_·g^−1^ iron. This result confirms the substantial oxidation of the iron in the pristine film samples after the thermomechanical process. More precisely, iron in fresh LLDPE.Fu15.MMT-Fe6.25 and LLDPE.Fu15.MMT-Fe3.75 was approximately twice and three times more oxidized, respectively, than in MMT-Fe, which agrees with Mössbauer spectroscopy analysis.

Moreover, the iron oxidation kinetics was slower in LLDPE.Fu15.MMT-Fe3.75 than in LLDPE.Fu15.MMT-Fe6.25 and did not reach equilibrium at the end of the experiment (two weeks). This might have been due to the larger thickness of LLDPE.Fu15.MMT-Fe3.75, owing to extrusion constraints at lab scale, which limited O_2_ diffusion to the core of the film and led to a lower apparent O_2_ adsorption capacity.

The adsorption capacity of the active films prepared here was in the same order of magnitude of those of previously published systems (Table 4). A calculation of the benefit of using these active films for food packaging applications is provided in Appendix B.

### 3.5. Oxygen and Water Vapor Barrier Properties of LLDPE/MMT-Fe Active Film Composite

**Figure 7:**
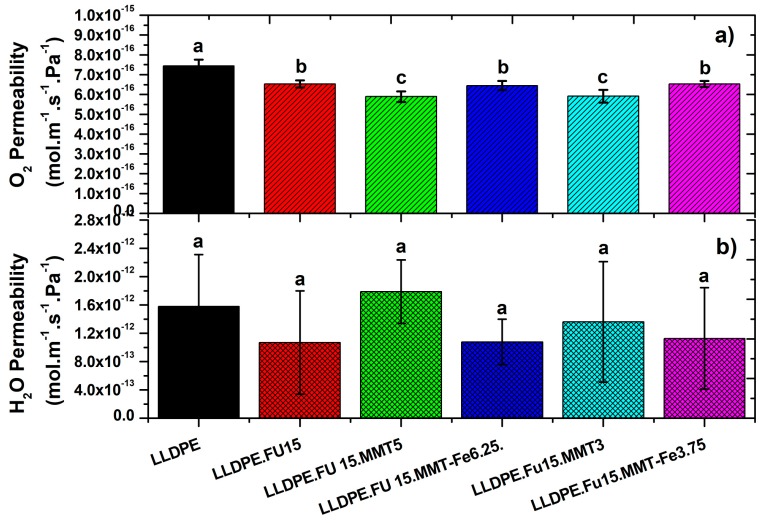
(**a**) Oxygen permeability and (**b**) water vapor permeability of (**a**) LLDPE film and LLDPE with compatibilizer and active films containing MMT-Fe with different contents (3.75% or 6.25%). The data are reported as mean ± standard deviation of four replicates. Bars with different letters represent significant difference (ANOVA test, *p*-value ≤ 0.05). Raw data corresponding to this figure has been uploaded at https://doi.org/10.5281/zenodo.3253103.

The oxygen permeability of the composite films at 23 °C and 0% RH conditions is shown in Figure 7a. The addition of PE-g-MA to the LLDPE reduced the permeability by about 15%. The addition of 3% or 5% MMT, producing an intercalated nanocomposite structure, further reduced the permeability by 20% compared with LLDPE. No significant difference in oxygen permeability was observed when MMT-Fe was added instead of MMT. Even if the active films displayed significant O_2_-scavenging properties (see previous paragraph), this scavenging effect did not impact the O_2_ permeability property in the conditions of measurement of the present work.

Also, the water vapor permeability was checked for all the films. No significant differences were detected among the different films (Figure 7b), also considering the relatively large experimental error bars. Further experiments are thus needed to confirm this result.

### 3.6. Modeling the Oxygen-Scavenging Activity

**Figure 8:**
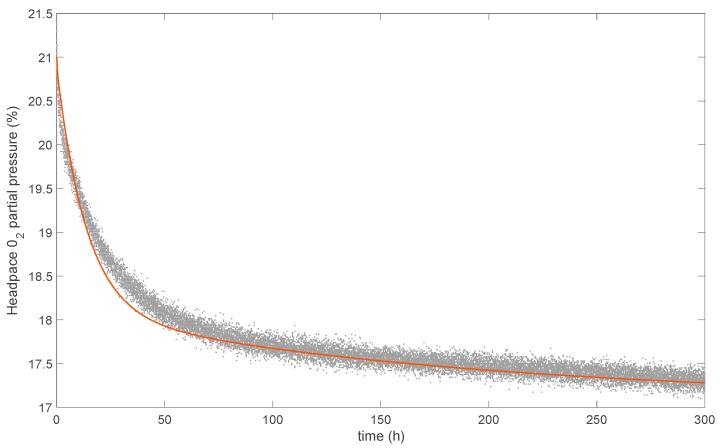
Comparison between simulated (red line) and experimental (grey dots) headspace–O_2_ depletion curves.

The reaction model developed in the previous work of Kombaya-Touckia-Linin et al. was here combined with a model describing O_2_ solubilization and diffusion in the polymer matrix. The parameters used for modeling O_2_ adsorption by LLDPE.Fu15.MMT-Fe6.25, calculated according to Equations (6), (7), (9), and (12), are listed in Table 5, while the comparison between experimental and simulated O_2_ depletion is shown in Figure 8. Good agreement was found between the experimental and modeled curves. It is worth noting that no additional parameters were required. Even with the simplified assumptions, the reaction–diffusion model was able to reproduce the observed data of O_2_ adsorption in an active nanocomposite, validating the O_2_ adsorption model previously developed for MMT-Fe.

**Figure 9:**
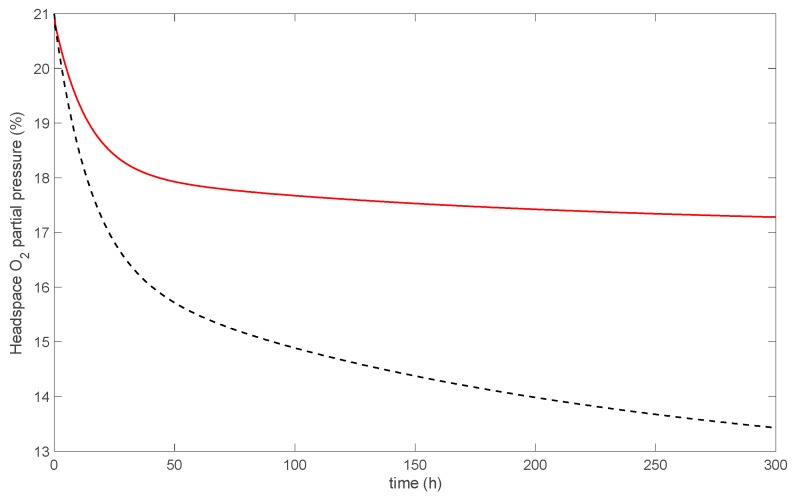
Simulated headspace O_2_ partial pressure: actual case, i.e., with oxidation during extrusion (red line), and theoretical case, i.e., with no oxidation during extrusion (black dashed line).

The O_2_-scavenging properties of the produced films were strongly impacted by the extrusion process, which induced substantial iron oxidation. As a comparison, a simulation was performed assuming that no iron oxidation occurs during extrusion (Figure 9). This “theoretical” case obviously produces higher O_2_ adsorption capacities, twice as much as actually measured. The production of such highly active O_2_-scavenging film will require a thorough optimization of both the synthesis of MMT-Fe active powder and the extrusion process leading to the nanocomposite film.

## 4. Conclusions

The oxygen-scavenging capacity of an MMT-Fe composite, designed as the active species for monolayer film applications, as well as its LLDPE nanocomposites was evaluated. The MMT-Fe contained Fe_1−x_B_x_ nanoparticles with an average aggregate diameter of ~57 nm deposited on the surface of MMT clay platelets. Active nanocomposites containing 6.25 or 3.75 wt % of MMt-Fe were manufactured by extrusion with LLDPE, showing adsorption capacities two times lower than that of pristine MMT-Fe. This decrease in adsorption capacity was caused by the oxidation of the Fe_1−x_B_x_ nanoparticles during the extrusion process, as assessed by Mössbauer spectroscopy. The active films containing 3.75 and 6.25 wt % were able to uptake between 31 ± 2 and 55 ± 9 mg O_2_.g^−1^ iron, respectively, while MMT-Fe had an adsorption capacity of 122 mg O_2_.g^−1^ iron at 20 °C and 100% RH. The reaction–diffusion model was able to reproduce the observed data of O_2_ adsorption. This study suggests that there is significant potential for the use of this new oxygen-scavenging composite, MMT-Fe, which, if included in packaging under the correct anoxic conditions, might help to extend the shelf life of oxygen-sensitive food products.

## Figures and Tables

**Table 1:** Labeling and composition of the active films prepared.

Sample *	Composition			
	LLDPE wt %	PE-g-MA wt %	MMT wt %	MMT-Fe wt %
**LLDPE**	100			
**LLDPE.Fu15**	85	15		
**LLDPE.Fu15.MMT3**	82	15	3	
**LLDPE.Fu5.MMT5**	80	15	5	
**LLDPE.Fu15.MMT-Fe3.75**	81.25	15		3.75
**LLDPE.Fu15.MMT-Fe6.25**	78.75	15		6.25

Abbreviations: LLDPE—linear low-density polyethylene; PE-g-MA—polyethylene-grafted maleic anhydride; MMT—montmorillonite. * The number after “Fu” stands for the wt % of PE-g-MA used in the formulation, the number after “MMT” for the wt % of MMT used in the preparation of iron-free reference samples, and the number after “MMT-Fe” for the wt % of MMT-Fe used in active film formulation.

**Table 2:** Thermal properties of LLDPE and LLDPE nanocomposites.

Sample	Heat of Fusion (J/g)	Tm (°C)	Crystallinity	Temp. Onset Degradation (°C)	Temp. Endset Degradation (°C)
LLDPE	104 ± 10	122.1 ± 0.5	35.8 ± 3.6	416 ± 9	475 ± 4
LLDPE.Fu15	104 ± 8	121.3 ± 1.2	35.7 ± 3.1	416 ± 2	457 ± 2
LLDPE.Fu15.MMT3	104 ± 5	121.9 ± 0.7	34.8 ± 0.9	416 ± 22	478 ± 19
LLDPE.Fu15.MMT5	99 ± 10	122.2 ± 1.0	31.3 ± 0.6	429 ± 9	480 ± 4
LLDPE.Fu15.MMT-Fe3.75	107 ± 2	121.6 ± 0.4	36.3 ± 0.7	419 ± 5	468 ± 3
LLDPE.Fu15.MMT-Fe6.25	102 ± 4	122.3 ± 1.2	34.5 ± 1.2	418 ± 7	466 ± 2

The data reported here were not significantly different (ANOVA test, *p*-value ≤ 0.05). Raw data corresponding to this figure are uploaded at https://doi.org/10.5281/zenodo.3253103. See supplementary material.

**Table 3:** ^57^Fe Mössbauer parameters at 5 K for samples MMT-Fe, fresh LLDPE.Fu15.MMT-Fe6.25, and aged LLDPE.Fu15.MMT-Fe6.25.

Sample	Component	δ (mm/s)	Δ (mm/s)	Γ (mm/s)	B_HF_ (T)	Area (%)
**MMT-Fe**	Fe_1−x_B_x_	0.08	−0.03	0.47	30.8	53
	Fe^2+^ para	1.25	3.03	0.66	-	6
	Fe^2+^ magn	1.21	−2.78	1.72	15.0	6
	Fe^3+^ oxide	0.37	−0.12	0.60	49.0	35
**Fresh LLDPE.Fu15.MMT-Fe6.25**	Fe_1−x_B_x_ type1	0.00	0.00	0.60	25.00	3
	Fe_1−x_B_x_ type2	0.24	0.00	0.86	-	8
	Fe^2+^ para	1.28	3.09	0.64	-	4
	Fe^2+^ magn	1.21	−2.07	1.14	19.45	10
	Fe^3+^ oxide type1	0.34	0.00	0.85	51.62	75
	Fe^3+^ oxide type 2	0.41	0.00	2.5	47.17	
**LLDPE.Fu15.MMT-6.25 after four months of oxidation**	Fe_1−x_B_x_ type1	0.00	0.00	0.60	24.55	3
	Fe_1−x_B_x_ type2	0.30	0.00	1.11	-	3
	Fe^2+^ para	1.30	3.04	0.56	-	3
	Fe^2+^ magn	1.21	−2	1.81	19.52	5
	Fe^3+^ oxide type1	0.37	0.00	1.27	50.76	86
	Fe^3+^ oxide type 2	0.41	0.00	2.5	46.16	

**Table 4:** Comparison with results reported in the literature.

Film	O_2_ Adsorption Capacity mg O_2_/g of Absorber	Ref.
PE.Fu15.MMT-Fe6.25 film	55 ± 2	This work
PE.Fu15.MMT-Fe3.75 film	31 ± 2	This work
Polymer with iron powder and additives (“SHELFPLUS”); masterbatch	26–48	[36]
O2Block1 (NanoBioMatters S.L., Paterna, Spain); masterbatch	>10–25	[37]
Cyclo-olefin bonded to a silicate backbone “ORMOCER1”; lacquer	90	[38]
Nanoscale iron in silicon matrix	>32 ± 80	[7]
Polyolefin films containing 10 wt % of iron modified kaolinite	5.7	[9]
Nanoscale iron powder	>68 ± 2	[7]
Nanoscale iron-MMT powder	122	[15]
Stoichiometric capacity of iron	394	[15]

**Table 5:** Parameter descriptions and values for numerical simulation of O_2_ adsorption for the PE.Fu15.MMT-Fe6.25 sample.

Symbol	Definition	Unit	Value	Source
L	Thickness of the nanocomposite sample	m	296 × 10−6	This work
A	Surface area of a single side of the nanocomposite film sample	$m^{2}$	0.1376	This work
$V_{HS}$	Volume of the headspace	$m^{3}$	458 × 10−6	This work
$x_{F_{e}}^{f}$	Mass fraction of iron in the sample	$\mathrm{kg}\left(\mathrm{iron}\right)\cdot \mathrm{kg}{\left(\mathrm{sample}\right)}^{-1}$	0.22	This work
$x_{F_{e}}$	Mass fraction of zerovalent iron	$\mathrm{kg}\cdot \mathrm{kg}{\left(\mathrm{iron}\right)}^{-1}$	0.11	This work
$x_{{\mathrm{Fe}(\mathrm{OH})}_{2}}$	Mass fraction of bivalent iron	$\mathrm{kg}\cdot \mathrm{kg}{\left(\mathrm{iron}\right)}^{-1}$	0.14	This work
$M_{F_{e}}$	Molar mass of iron	$\mathrm{kg}\cdot {\mathrm{mol}}^{-1}$	55.8 × 10−3	
$\rho_{f}$	Apparent density of the nanocomposite, taken as the corresponding value for the pure polymer (LLDPE)	$\mathrm{kg}\cdot m^{-3}$	918	[15]
$k_{1}$	Kinetic coefficient of Fe oxidation	$m^{3}\cdot {\;s}^{-1}\cdot {\mathrm{mol}}^{-1}$	2.4 × 10−6	[15]
$k_{2}$	$\mathrm{Kinetic}\;\mathrm{coefficient}\;\mathrm{of}\;{\mathrm{Fe}(\mathrm{OH})}_{2}$ oxidation	$m^{3}\cdot {\;s}^{-1}\cdot {\mathrm{mol}}^{-1}$	3.2 × 10−5	[15]
$n_{1}$	Apparent stoichiometric coefficient of Fe oxidation	$\mathrm{mol}\cdot {\mathrm{mol}}^{-1}$	0.19	[15]
$n_{2}$	$\mathrm{Apparent}\;\mathrm{stoichiometric}\;\mathrm{coefficient}\;\mathrm{of}\;{\mathrm{Fe}(\mathrm{OH})}_{2}$ oxidation	$\mathrm{mol}\cdot {\mathrm{mol}}^{-1}$	0.40	[15]
$D_{O_{2}}$	Apparent O_2_ diffusivity in the nanocomposite, taken as the corresponding value for the pure polymer (LLDPE)	$m^{2}\cdot s^{-1}$	1.68 × 10−11	[39]
$k_{H}$	O_2_ solubility in the nanocomposite material	mol·Pa−1· m−3	3.54 × 10−6	[40]
Bi	Biot number	dimensionless	1 × 105	This work
R	Ideal gas constant	$J{.\mathrm{mol}}^{-1}\cdot K^{-1}$	8.314	
T	Temperature of the experiment	K	293.15	This work

**Figure A1:**
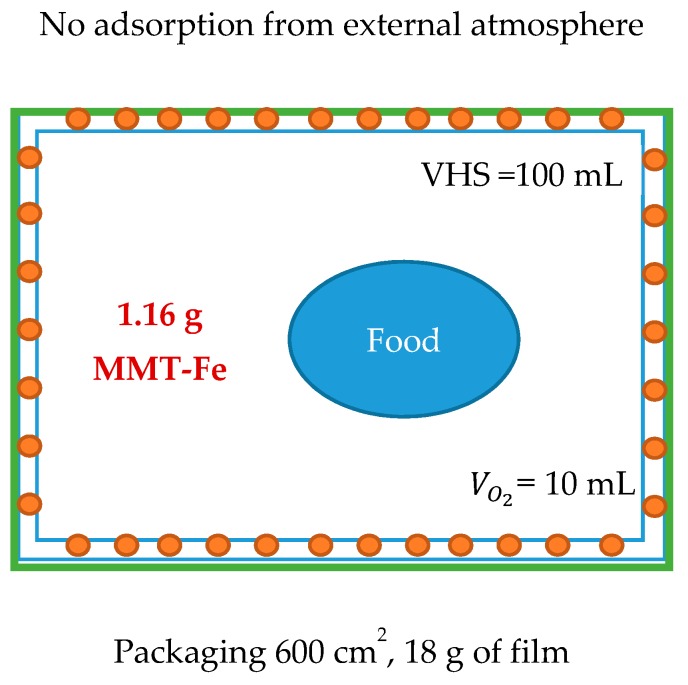
Schematic representation of adsorption capacity compatibility with food packaging.

## Supplementary Materials

Raw data of Table 2 and Figure 7 are available online at https://www.mdpi.com/2079-4991/9/9/1193/s1: and https://doi.org/10.5281/zenodo.3253103.

## Appendix A

More details about the initial and boundary conditions of the diffusion–reaction model developed in the present work are provided hereafter.

**Initial and boundary conditions.** At initial time $t_{0}$, uniform concentrations $C_{\mathrm{Fe}}\;\left(t_{0}\right)$ and $C_{{\mathrm{Fe}(\mathrm{OH})}_{2}}\left(t_{0}\right)$ were considered and computed according to Equation (7), renamed hereafter as Equation (A1):

(A1) $$\begin{array}{c} C_{\mathrm{Fe}}\;\left(t_{0}\right)=\frac{\rho_{f}\;x_{F_{e}}^{f}\;x_{F_{e}}}{M_{F_{e}}}\\ C_{{\mathrm{Fe}(\mathrm{OH})}_{2}}\left(t_{0}\right)=\frac{\rho_{f}\;x_{F_{e}}^{f}\;x_{{\mathrm{Fe}(\mathrm{OH})}_{2}}}{M_{F_{e}}} \end{array}$$

where $x_{F_{e}}^{f}$(kilograms of iron per kilogram of sample) is the mass fraction of iron in the sample, $x_{F_{e}}$ and $x_{{\mathrm{Fe}(\mathrm{OH})}_{2}}$(kilograms per kilogram of total iron) are the mass fraction of zerovalent and divalent iron, respectively, $M_{F_{e}}$(kg·mol^−1^) is the molar mass of iron, and $\rho_{f}$ (kg·m^−3^) is the apparent density of the nanocomposite. A null oxygen concentration, $C_{O_{2}}\left(t_{0}\right)=0$, was assumed.

Focusing on the O_2_ adsorption by an active nanocomposite film, the material was surrounded by a unique headspace, and the external conditions at upper and lower surfaces were equal. The O_2_ molar flow $\varphi_{L/2}$ (mol·s^−1^) at the upper headspace–film interface $\Gamma_{L/2}$ is given in Equation (A2):

(A2) $$\varphi_{L/2}=k\;A\;\left(C_{0_{2},\;\mathrm{HS}}-C_{0_{2},\;\mathrm{HS},L/2}\right)$$

where k (m·s^−1^) is the external mass transfer coefficient at the upper and lower surfaces $\Gamma_{L/2}$ and $\Gamma_{-L/2}$; A (m^2^) is the surface area of the headspace–film interface, which was assumed to be identical for the upper and lower surfaces; and $C_{0_{2},\;\mathrm{HS}}$ (mol·m^−3^) and $C_{0_{2},\;\mathrm{HS},i}$ (mol·m^−3^) are, respectively, the O_2_ concentration in the headspace and at the vicinity of the composite surface.

From the ideal gas law, Equation (A2) can be rewritten as

(A3) $$\varphi_{L/2}=k\;\frac{A}{\mathrm{RT}}\;\left(p_{0_{2},\;\mathrm{HS}}-p_{0_{2},\;\mathrm{HS},L/2}\right)$$

where T (K) is the temperature, R is the ideal gas constant, $p_{0_{2},\;\mathrm{HS}}$ (Pa) and $p_{0_{2},\;\mathrm{HS},i}$ (Pa) are the O_2_ partial pressure in the headspace and at the vicinity of the composite surface, respectively.

According to Henry’s law, assuming that the O_2_ is at a thermodynamic equilibrium between the composite and the headspace, the concentration $C_{O_{2}}\left(t,x=L/2\right)$ (mol·m^−3^) of dissolved O_2_ at the composite surface is proportional to $p_{0_{2},\;\mathrm{HS},L/2}$, the O_2_ partial pressure in the vicinity of the composite surface. The O_2_ flow $\varphi_{L/2}$ at the upper headspace–film interface $\Gamma_{L/2}$ becomes

(A4) $$\varphi_{L/2}=k\;\frac{A}{\mathrm{RT}}\;\left(p_{0_{2},\;\mathrm{HS}}-\frac{C_{O_{2}}\left(t,x=L/2\right)}{k_{H}}\right)$$

where k_H_ (mol·Pa^−1^·m^−3^) is the O_2_ solubility in the composite material. In the same way, the O_2_ flow $\varphi_{-L/2}$ at the lower headspace–film interface $\Gamma_{-L/2}$ is described by Equation (A5):

(A5) $$\varphi_{-L/2}=k\;\frac{A}{\mathrm{RT}}\;\left(p_{0_{2},\;\mathrm{HS}}-\frac{C_{O_{2}}\left(t,x=-L/2\right)}{k_{H}}\right).$$

Finally, the boundary conditions can be written as

(A6) $$\begin{array}{l} D_{O_{2}}\;\frac{\partial C_{O_{2}}\left(t,x\right)}{\partial x}=\frac{\varphi_{\frac{L}{2}}}{A}=\frac{k}{\mathrm{RT}}\;\left(p_{0_{2},\;\mathrm{HS}}-\frac{C_{O_{2}}\left(t,x\right)}{k_{H}}\right)\;\mathrm{at}\;x=\frac{L}{2}\;\mathrm{and}\;\forall t\geq 0\\ D_{O_{2}}\;\frac{\partial C_{O_{2}}\left(t,x\right)}{\partial x}=\frac{\varphi_{-\frac{L}{2}}}{A}=\frac{k}{\mathrm{RT}}\;\left(p_{0_{2},\;\mathrm{HS}}-\frac{C_{O_{2}}\left(t,x\right)}{k_{H}}\right)\;\mathrm{at}\;x=-\frac{L}{2}\;\mathrm{and}\;\forall t\geq 0 \end{array}$$

## Appendix B

In order to verify the possible advantage of using active films prepared in the present work as O_2_ scavengers for food packaging applications, their theoretical adsorption capacity in a real situation was estimated. We considered a food/packaging system stored at ambient temperature, with a packaging headspace of 100 mL. We considered the adsorption of O_2_ by the food as well as its permeation through the packaging to be negligible. In that condition, including about 1 g of MMT-Fe powder in the headspace polymer would permit a decrease of the volume of O_2_ from 21 to 1.5 mL. For the same amount of MMT-Fe but embedded in the bulk polymer (active film), considering its level of oxidation after processing and that the film, in the ideal case, would be protected from the outside atmosphere by a barrier layer before use, the decrease of O_2_ in the headspace would be about half of the initial content (10.5 mL) (Figure A1). In such conditions, the active packaging would be much less efficient than MMT-Fe used alone in the headspace and insufficient for acceptable removal of O_2_ from the packaging. However, such a film could have a positive impact when used in combination with other methods, such as modified-atmosphere packaging (e.g., N_2_ flush) for the adsorption of residual amounts of O_2_ in the packaging atmosphere.
